# Drug resistance profile of *Mycobacterium tuberculosis* in China: update until 2024

**DOI:** 10.3389/fmicb.2025.1697490

**Published:** 2025-12-09

**Authors:** Yannan Xu, Sixuan Liu, Jiaxiong Zheng, Jianxiong Lin, Liwei Gi, Qiaocheng Chang

**Affiliations:** 1 School of Public Health, Shantou University, Shantou, China; 2 School of Basic Medicine and Public Health, Jinan University, Guangzhou, China; 3 Shantou Tuberculosis Prevention and Control Institute, Shantou, China; 4 State Key Laboratory for Zoonotic Diseases, Key Laboratory for Zoonosis Research of the Ministry of Education, Institute of Zoonosis, and College of Veterinary Medicine, Jilin University, Changchun, China

**Keywords:** *Mycobacterium tuberculosis*, drug resistance, gene mutation, molecular epidemiology, mutation profile

## Abstract

**Background:**

The situation of drug-resistant tuberculosis in China remains serious and complex. The majority of the study data are still derived from the 2,207 national survey of drug-resistant tuberculosis. In this study, we aimed to comprehensively characterize the prevalence of *Mycobacterium tuberculosis* (MTB) in China and update the catalogue of drug-resistant mutations while accounting for geographic variability.

**Materials and methods:**

This study analyzed *Mycobacterium tuberculosis* (*M. tuberculosis*) isolates collected from 27 provinces, municipalities, and autonomous regions across China. All strains were analyzed for resistance to isoniazid, rifampicin, streptomycin, ethambutol, pyrazinamide, and quinolones based on the results of phenotypic drug sensitivity tests. The spatial and temporal distribution characteristics of drug-resistant strains were assessed based on the geographic origin and collection time of the isolates. The association between mutations and drug resistance was evaluated using mutation rates, positive predictive values, chi-square or Fisher’s exact test *p*-values, and 95% confidence intervals.

**Results:**

55,388 MTB strains collected from 2002 to 2024 were analyzed, among which 15,078 were drug-resistant, including 7,848 multidrug-resistant strains. The resistance rates for INH, RFP, SM, EMB, PZA, and QS were 27.67, 25.33, 11.55, 6.19, 8.63, and 20.63%, respectively. Regional distribution patterns revealed that the eastern and western regions had the highest number of strains, but relatively low resistance rates. There was a low inflection point in 2019 for the resistance rates of all drugs except INH, whose resistance rate continued to increase after 2017. A total of 754 non-synonymous mutations were identified, with the highest mutation rates observed in INH (32.91%), RIF (28.98%), and QS (14.47%). The dominant mutation sites were *katG* (315AGC → ACC), *rpoB* (531TCG → TTG), and *gyrA* (94GAC → GGC), respectively. In addition, 96 newly detected mutations potentially associated with drug resistance were identified, including *ahpC* (11CCG → CCG) and *pncA* (226ACT → CCT). Combined mutations were most frequently observed in *rpoB* + *rpoB*, *katG* + *katG*, and *katG* + *inhA*, with other double-mutation combinations also being predominant.

**Conclusion:**

Our analysis demonstrates that drug-resistant tuberculosis remains a serious challenge in China. Newly identified resistance-conferring mutations should be prioritized and integrated with the specific epidemiological characteristics of DR-TB in China to support the development and implementation of rapid diagnostic technologies.

## 1 Introduction

*Mycobacterium tuberculosis* (MTB) is the pathogen responsible for tuberculosis (TB). The World Health Organization (WHO) aims to eliminate the global tuberculosis epidemic by 2035. However, the emergence of drug-resistant tuberculosis (DR-TB) has posed even greater challenges to resource-poor, high TB-burden countries due to its prolonged treatment duration, significant drug side effects, and increased risk of death (World Health Organization, 2024; Treatment Action Group, Stop TB Partnership, 2025). China currently faces a severe burden of DR-TB. In 2023, the number of new multidrug-resistant/rifampicin-resistant tuberculosis (MDR/RR-TB) cases ranked fourth globally, accounting for 7.3% of the global total (World Health Organization, 2024). Additionally, due to disruptions caused by the COVID-19 pandemic in 2019 (WHO, 2025), the epidemiological trends of DR-TB in China have become more complex. To accurately guide tuberculosis prevention and control efforts and narrow the gap between the detection and treatment of drug-resistant cases, it is essential to update the epidemiological characteristics of MTB in a timely manner (Wan et al., 2020; Mohamed et al., 2021). This involves investigating whether current research can identify genes or loci with potential as drug-resistant targets, thereby deepening our understanding of the regional distribution patterns of drug-resistant tuberculosis.

Phenotypic drug susceptibility testing (DST) remains the gold standard for detecting drug resistance in MTB. However, such methods are cumbersome and time-consuming (7–56 days) and require expensive and complex laboratory capabilities, making them difficult to implement and apply in countries with a high burden of DR-TB (Bwanga et al., 2009). The WHO recommends the use of rapid molecular tests or sequencing technologies for drug resistance testing of MTB (World Health Organization, 2022). For example, nucleic acid-based molecular diagnostic techniques, such as polymerase chain reaction (PCR), whole-genome sequencing (WGS), and GeneXpert, have significantly simplified and accelerated the diagnosis of DR-TB (Shinnick et al., 2015; Boehme et al., 2010). However, the sensitivity and specificity of these methods may vary depending on the region of use (Ferro et al., 2013). Therefore, a comprehensive understanding of the epidemiological characteristics of drug-resistant MTB and the genes associated with drug resistance in a particular region is crucial for overcoming regional limitations in drug resistance.

Acquired antibiotic resistance in MTB is primarily characterized by mutations in genes encoding drug targets or drug-activating enzymes. This enables bacteria to survive under external pressures such as drug exposure, thereby leading to DR-TB (Wan et al., 2020; Gygli et al., 2017). In 2021, the WHO compiled a catalog of resistance-associated mutations to serve as a global standard for interpreting molecular data to predict drug resistance (Walker et al., 2022). For instance, mutations in *katG* (particularly at codon 315) and the *inhA* promoter region (e.g., at position −15) are strongly associated with resistance to isoniazid (INH) (Liu, 2024; Ando et al., 2014). The Rifampicin (RIF) resistance-determining region (RRDR) is widely used to identify rifampicin-resistant strains (Unissa et al., 2016). Resistance to Quinolones (QS) often involves mutations in *gyrA* and *gyrB* (Kocagöz et al., 1996), while Streptomycin (SM) resistance is commonly linked to mutations in *rpsL* and *rrs* (Maningi et al., 2018). The *embB*306 is the primary mutation site in ethambutol-resistant (EMB) MTB (Mokrousov et al., 2002). Additionally, unlike the relatively concentrated resistance genes and sites mentioned above, pyrazinamide resistance (PZA) can be caused by many individually infrequent mutations dispersed across *pncA* (Köser et al., 2020). However, due to specific issues such as social pressure and disease burden in different countries, complex or additional resistance mutations may arise (Pei et al., 2024), so the specific situation in China requires further detailed analysis.

Currently, the most recent strain data for analyzing the drug resistance epidemiology of tuberculosis in China still originate from the 2007 National Drug-Resistant Tuberculosis Survey (Zhao et al., 2012). Such nationwide surveys are typically targeted, and regions experiencing economic growth may receive more opportunities for reporting. Additionally, in recent years, factors such as the COVID-19 pandemic and antibiotic misuse have slowed the previously declining trend of tuberculosis incidence in China, further contributing to a complex drug resistance landscape (Liu et al., 2022). Therefore, this study systematically analyzed clinical MTB strains isolated from various regions across China over the past two decades. The analysis encompassed not only strains from nationally designated sentinel sites but also incorporated data from spontaneously conducted regional DR-TB surveys at the prefectural level. It obtained and integrated the drug resistance profiles and mutation patterns of drug resistance-associated genes from these strains. This study provides scientific reference for updating the epidemiological status of drug-resistant tuberculosis in China, broadening the scope of regional drug-resistant tuberculosis investigations, and exploring the mutation characteristics of drug-resistant genes. It aims to advance the optimization of precision control strategies for drug-resistant tuberculosis.

## 2 Materials and methods

### 2.1 Data sources

As of September 1, 2024, we collected data on drug resistance in MTB in China from eligible studies retrieved from PubMed, Web of Science, China National Knowledge Infrastructure (CNKI), Wanfang Data, and VIP Database (Supplementary Table 1). Studies were included if the strains in the literature originated from China, and the results must include DST resistance results, molecular detection resistance results, resistance gene mutation types, and mutation site information. Studies were excluded if they did not perform DST or lacked information on resistance gene mutations, or if multiple publications described the same sample set—in such cases, the study with the largest sample size or the most recent publication date was selected.

### 2.2 Definitions related to drug resistance

INH^R^, RIF^R^, SM^R^, QS^R^, PZA^R^, and EMB^R^ are defined as MTB resistant to INH, RIF, SM, QS, PZA, and EMB, respectively. INH^S^, RIF^S^, SM^S^, QS^S^, PZA^S^, and EMB^S^ are defined as MTB sensitive to INH, RIF, SM, QS, PZA, and EMB, respectively. DR-TB is defined as tuberculosis caused by MTB resistant to one or more anti-tuberculosis drugs. MDR-TB is defined as tuberculosis resistant to at least two first-line anti-tuberculosis drugs, INH and RIF (World Health Organization, 2022). To mitigate potential bias in drug resistance data arising from heterogeneous detection strategies, this study categorizes references into single-drug detection studies (S strategy) and multi-drug detection studies (M strategy) based on detection approach. A single-drug detection study is defined as one where only one target drug is present and detected, such as assessing resistance to either RFP or INH alone. Multi-drug detection studies are defined as those where two or more target drugs are present, such as detecting resistance to both RFP and INH or to multiple drugs including RFP, INH, and SM.

### 2.3 Identifying drug resistance-associated gene mutations in bacterial strains

This study integrated the first and second editions of the WHO-published catalogues of drug-resistant-associated gene mutations in MTB and the pulmonary tuberculosis drug resistance database to identify a set of candidate genes and corresponding promoter sequences with a higher likelihood of being associated with drug resistance for each drug (Supplementary Table 7) (Walker et al., 2022; Sandgren et al., 2009; World Health Organization, 2023). Tier 1 indicates association with drug resistance; Tier 2 indicates association with intermediate drug resistance. After extracting all drug resistance data from the included literature, we first identified synonymous mutations that do not confer drug resistance, followed by mutations potentially associated with drug resistance (including single mutations and combined mutations). The phenotypic and molecular drug resistance identification results were compared to determine the association between each gene or region and drug resistance.

### 2.4 Criteria for determining drug resistance-associated mutations

Based on the standards established by Köser et al. (2020), using ORs, PPVs, *p*-values, and CIs as reference points, a mutation is considered to be associated with resistance if it is observed on at least five occasions, the lower bound of the 95% CI for PPV is at least 0.25, the OR is at least 1, and the *p*-value is significant. If the variant occurs only in susceptible isolates or appears solely in susceptible isolates, it is considered unrelated to resistance. If the mutation never occurred alone in susceptible isolates and was not exclusive to susceptible isolates, it was rated as of uncertain significance. Since PZA resistance arises from multiple rare mutations scattered across *pncA*, criteria were relaxed for pyrazinamide to avoid excessive exclusion of these mutations. A mutation is classified as associated with resistance if it occurs in at least two resistant isolates in *pncA* with a PPV of at least 50%. In contrast, mutations with a PPV below 40% (and an upper 95% CI below 75%) are classified as unrelated to resistance. Furthermore, to distinguish statistically significant resistance genes by importance, we defined high-confidence mutations as those explicitly documented in public databases (such as WHO-UCN-TB and PhyResSE) or confirmed through at least one independent experimental study (e.g., gene knockout, complementation assays) as being associated with drug resistance. All other newly identified mutations are classified as low-confidence mutations, requiring additional validation through bioinformatics techniques to establish their association with drug resistance.

### 2.5 Data processing and statistical analysis

Microsoft Excel 2024 was used for the organization and extraction of drug resistance-related information; R 4.5.1 was employed for data visualization and batch computation of odds ratios, positive predictive values, and confidence intervals. IBM SPSS Statistics (version 26.0; IBM Corp., Armonk, NY, USA) was used to perform chi-square and Fisher’s exact tests according to the sample size, as well as multivariate regression analysis. A *p* < 0.05 was considered statistically significant.

## 3 Results

### 3.1 Basic information on included strains

**Figure 1:**
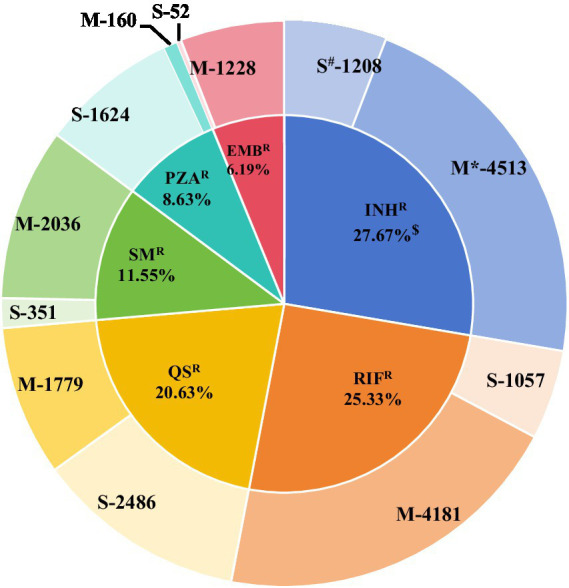
Distribution of 15,078 resistant strains. * Group M indicates the frequency of resistant strains obtained through the M-strategy test. ^#^ Group S indicates the frequency of resistant strains obtained through the S-strategy. ^$^ Frequency of drug resistance = frequency of drug resistance/total frequency of drug resistance. Here, the total frequency of occurrence of drug resistance in the 15,078 resistant strains is 20,675.

A total of 55,388 MTB collected between 2002 and 2024 were included in the final analysis. Among these, 18,781 (33.90%, 18,781/55,388) were tested using the S-strategy, while 36,607 (66.09%, 36,607/55,388) were tested via the M-strategy. Overall, 15,078 DR-TB strains were identified, including 7,848 MDR-TB strains, which accounted for 17.01% (7,848/46,138) of all isolates. Among the DR-TB strains, resistance to first-line anti-tuberculosis drugs—INH^R^(27.67%) and RFP^R^(25.33%) —and the second-line drug QS^R^(20.63%) was frequently observed. In contrast, SM^R^, PZA^R^, and EMB^R^ occurred at relatively lower frequencies (Figure 1; Supplementary Table 8).

### 3.2 Regional distribution characteristics of DR-TB strains

**Figure 2:**
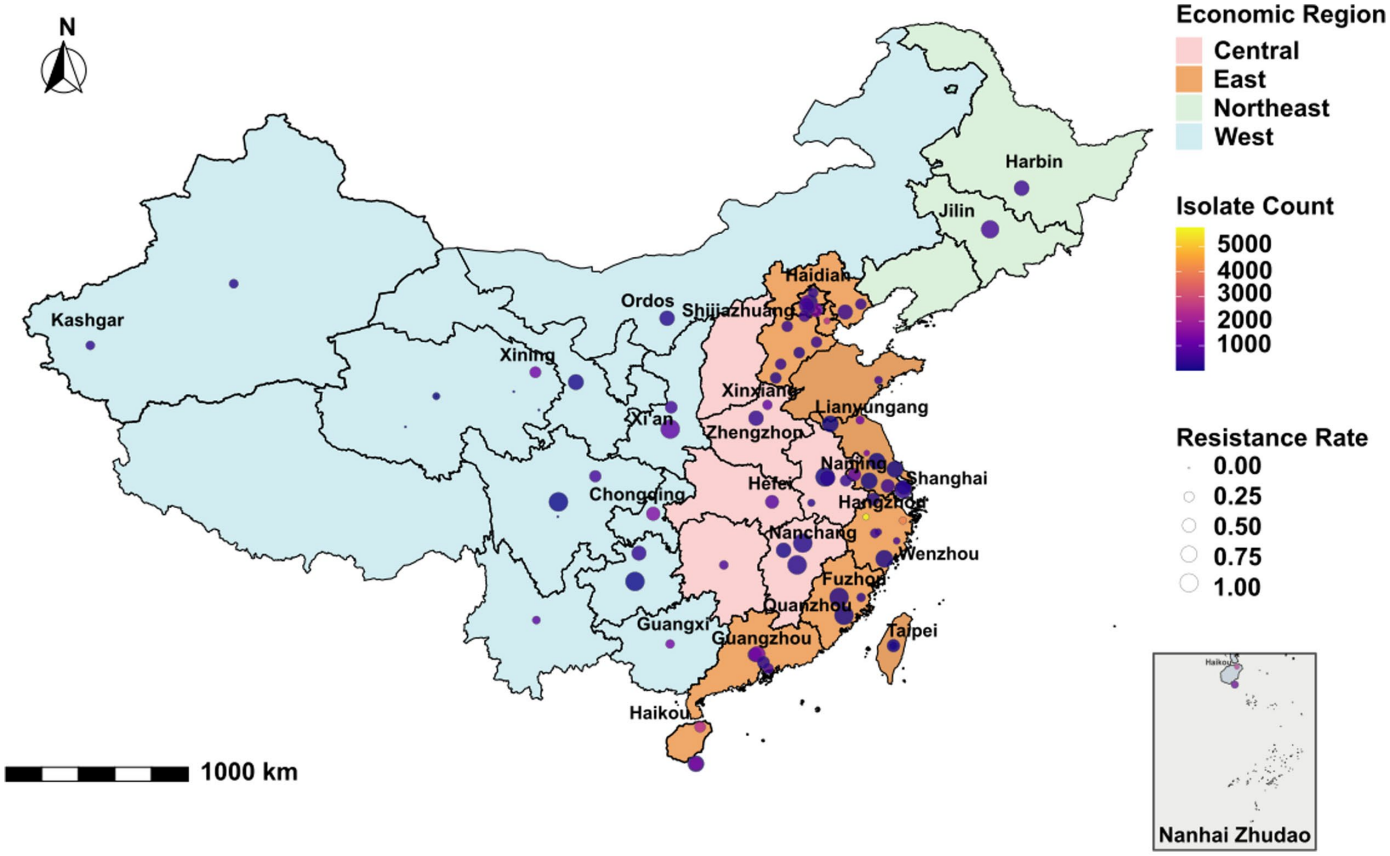
Geographical distribution of the included MTB isolates in China. Strains without precisely defined geographical origins within China were excluded from the analysis.

A total of 55,388 MTB strains were collected from 21 provinces, 4 municipalities, and 2 autonomous regions across China (Supplementary Table 2). According to China’s economic regional classification, the distribution of strain origins, in descending order, was as follows: the Eastern region (39,453), the Western region (9,992), the Central region (4,408), and the Northeastern region (1,360). The drug resistance rates, ranked from highest to lowest, were as follows: Northeastern region (74.49%), Central region (38.36%), Western region (36.95%), and Eastern region (21.69%) (Table 1). The national distribution and geographical origins of the collected strains are shown in Figure 2.

**Table 1:** Number of MTB strains by economic region in China.

Economic region	MTB strains^*^	Resistant strains^*^	Resistance rate
Eastern region	39,453	8,557	21.69%
Western region	9,992	3,692	36.95%
Central region	4,408	1,691	38.36%
Northeastern region	1,360	1,013	74.49%
Total	55,213	14,953	–

^*^Unspecified area strains have been eliminated.

### 3.3 Temporal distribution characteristics of drug-resistant strains

**Figure 3:**
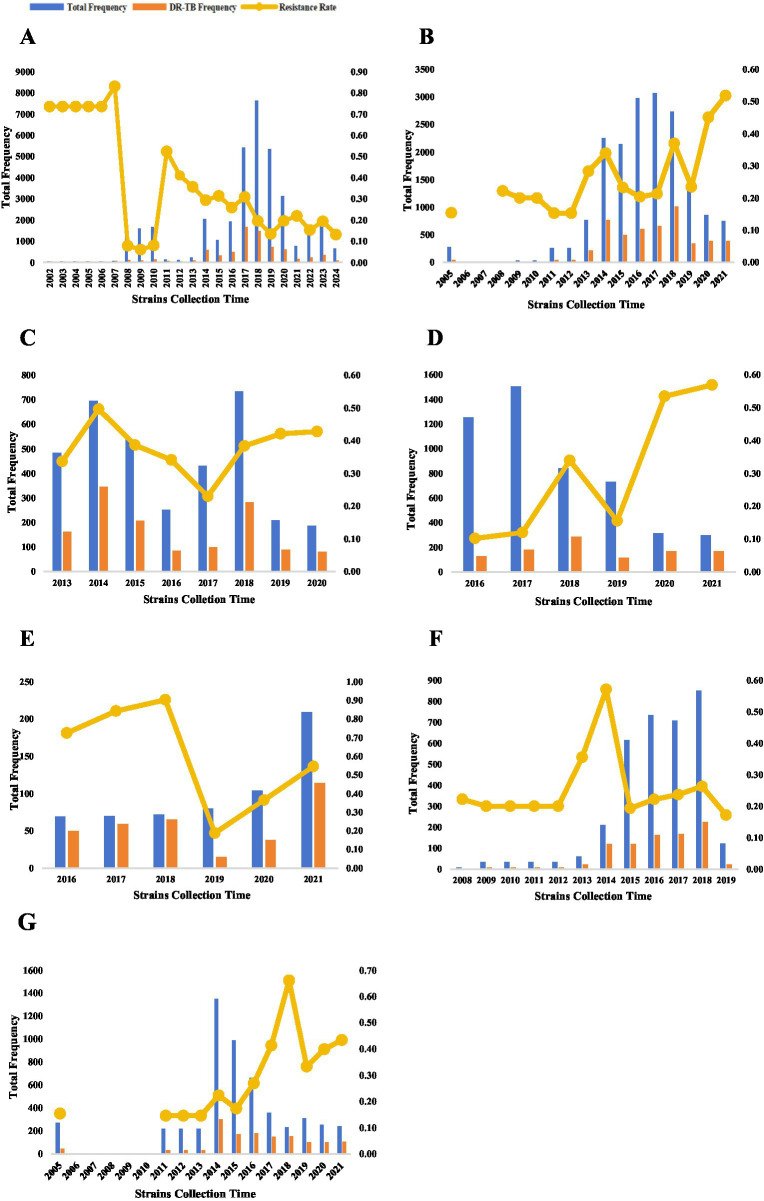
Temporal distribution of MTB strains included in the analysis **(A–G)**. The temporal distribution of MTB strains included in the analyses (continued). The temporal distribution of MTB strains detected by the M-strategy **(A)**, the temporal distribution of all MTB strains detected by the S-strategy **(B)**, and the temporal distribution of MTB strains detected by the S-strategy for INH^R^, RIF^R^, SM^R^, PZA^R^, and QS^R^ **(C–G)**. Temporal analyses were not performed because of the severe underrepresentation of MTB strains detected by the S-strategy for EMB^R^.

MTB strains included in this study via the M-strategy were collected between 2002 and 2024 (Supplementary Table 3). The number of MTB isolates generally showed an increasing trend until 2019, after which a decline was observed. Concurrently, although the overall drug resistance rate exhibited a downward trend, transient upward inflection points were noted in 2014, 2016, 2019, and 2022 (Figure 3A). Strains included via the S-strategy were collected over varying time periods (Supplementary Table 3). The collection of MTB isolates was primarily concentrated between 2014 and 2018, with a substantial decrease in the number of strains collected after 2019 (inclusive). Furthermore, compared to the results from the M-strategy, the drug resistance rate remained relatively high (20%–50%) for most of the period and gradually increased to its peak following the COVID-19 pandemic in 2019 (Figure 3B). Furthermore, a temporal analysis of individual anti-tuberculosis drugs based on the S-strategy revealed that the number of MTB isolates for INH, RIF, and QS agents with higher resistance rates gradually decreased after the peak period. In contrast, the number of isolates for SM, which had a lower resistance rate, showed a gradual increase. With the exception of INH, the resistance rates for RIF, QS, SM, and PZA all reached high-value inflection points in 2018, followed by low-value inflection points in 2019 (Figures 3C–G).

### 3.4 Synonymous mutations in candidate drug-resistance genes

A total of 79 synonymous type mutations were identified in 13 candidate genes and regions in this study (Supplementary Table 4). Although it is generally accepted that synonymous mutations are not associated with MTB resistance, we found that of the 79 synonymous mutations, 60.76% (48/79) occurred only in resistant strains, 34.18% (27/79) occurred only in sensitive strains, and the remaining four mutations occurred in both resistant and sensitive strains. Among the 13 candidate genes and regions carrying synonymous mutations, *rpsA* exhibited the highest mutation frequency (39.69%). Furthermore, the *rpsA* 636 CGA → CGC (Arg → Arg) mutation occurred at a higher frequency in PZA^R^ strains than in PZA^S^ strains, with a PPV as high as 66.02% (95% CI: 56.03–75.06%). None of the known synonymous mutations in *katG*, *rpoB*, *ahpC*, ndh, *kasA*, *gyrA*, *gyrB*, *pncA*, or *rpsA* independently occurred in the corresponding DR-TB strains. In contrast, six independent mutations in inhA, *rrs*, and *rpsL* were found exclusively in inhA-resistant, *rrs*-resistant, and *rpsL*-resistant strains, respectively. Additionally, the candidate gene *rpoB*—typically associated with RIF^R^—exhibited a 1,075 GCT → GCC (Ala→Ala) mutation in both INH^R^ and INH^S^ strains, with a higher frequency observed in INHR strains. Further integration of mutation information from the original strains carrying these synonymous mutations revealed that mutations at *katG* 440, 375, 295, 582, ndh 226, 107, *kasA* 139, and *gyrB* 428 did not occur independently but were instead accompanied by concurrent mutations at other sites or in other genes.

### 3.5 Characterization of mutations in candidate drug-resistance genes in MTB strains

**Figure 4:**
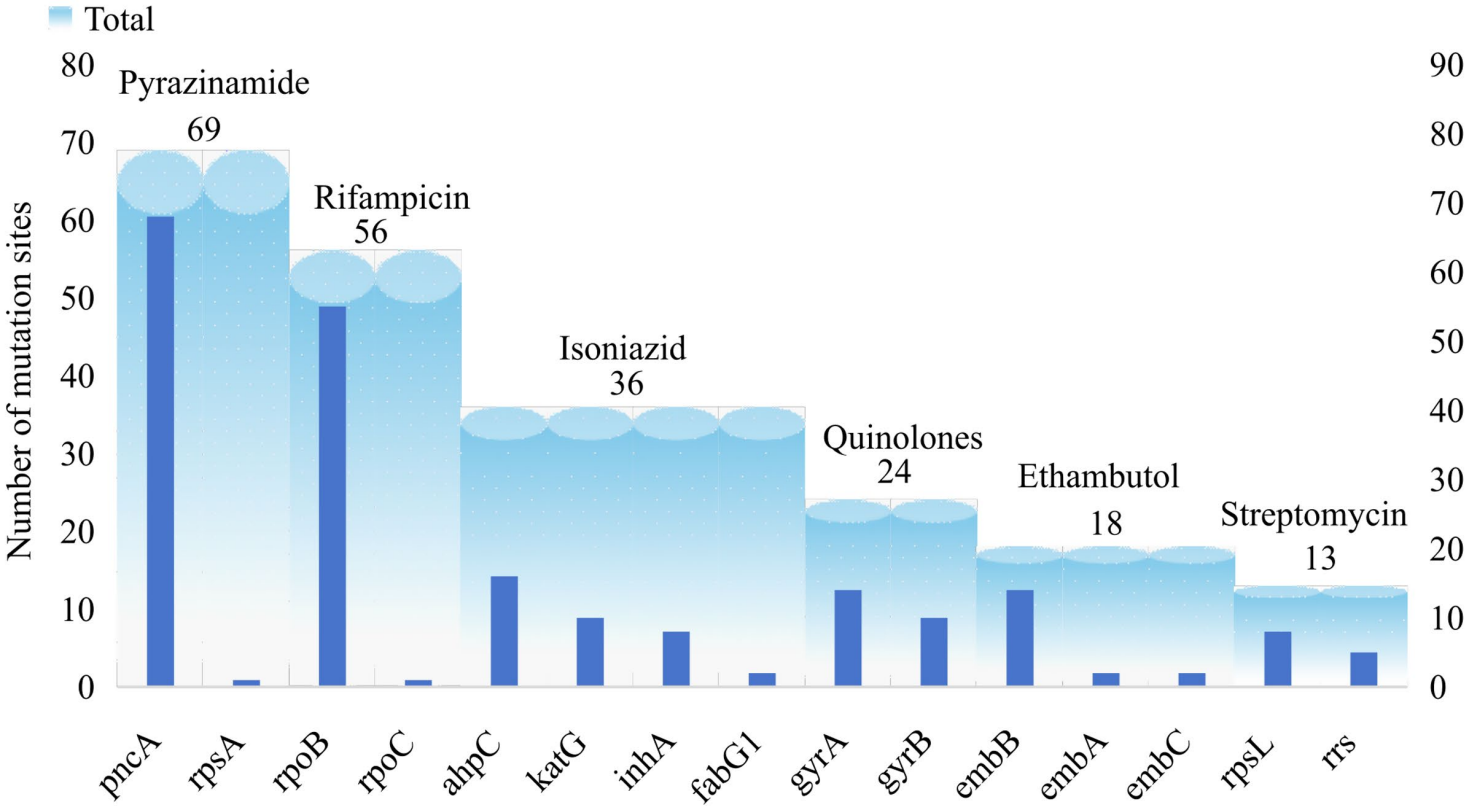
Distribution of resistance-associated mutation sites of candidate genes.

The catalogue of drug resistance gene mutations in MTB strains included in this study is shown in Supplementary Table 5. A total of 740 gene mutations were identified across 55,388 MTB strains, with point mutations being the predominant type. Based on the criteria for determining the association between gene mutations and drug resistance, 216 (29.19%) of the 740 mutations were found to be associated with drug resistance. With the exception of the candidate gene *kasA*, all other candidate genes exhibited mutations linked to drug resistance. Following comparison with public databases and validation experiments, we have identified 101 high-confidence mutations, which are highlighted in Supplementary Table 5. It should be noted, however, that this does not imply other novel mutations are insignificant; rather, further biological evidence is required to validate the association of these site mutations with drug resistance. Among these, 69 mutations in the candidate genes *pncA* and *rpsA* were associated with pyrazinamide resistance (PZA^R^), 56 mutations in *rpoB* and *rpoC* were associated with rifampicin resistance (RIF^R^), and 36 mutations in *ahpC*, *katG*, inhA, and *fabG1* were associated with isoniazid resistance (INH^R^). The number of mutation sites in the remaining candidate genes is shown in Figure 4.

A total of 216 candidate mutations associated with drug resistance were identified with an overall mutation rate of 98.51%. Among these, mutations related to PZA^R^ as defined by relaxed criteria accounted for 2.64%, while the remaining candidate genes accounted for 95.87% (Supplementary Table 5). Although the candidate genes associated with PZA^R^ exhibited the highest number of mutated sites, these mutations were scattered across the genome. The predominant mutations included 226ACT → CCT, 395GGT → GCT, and 29CAG → CCG, all of which were exclusively detected in PZA^R^ strains. For INH^R^, mutations were primarily identified in *katG*, inhA, and *ahpC*. In *katG*, the main mutations were 315AGC → ACC and 463CGG → CTG; in inhA, the predominant mutation was -15C → T; and in *ahpC*, the primary mutation observed was 11CCG → CCG. Regarding RIF^R^, the *rpoB* gene primarily exhibited the 531TCG → TTG mutation. For QS^R^, the main mutations occurred in *gyrA* at codon 94 GAC → GGC and in *gyrB* at codon 499AAC → AAG. In relation to EMB^R^, the *embB* gene mainly harbored the 306ATG → GTG mutation. For SM^R^, the most frequent mutation was 43AAG → AGG in *rpsL*, while mutations in *rrs* were predominantly 514A → C and 1401A → G. The major mutation sites in each candidate gene are summarized in Table 2.

**Table 2:** Main mutation sites of candidate genes.

Drug	*Gene*	Locus	Codon/nucleotide mutation	Total
INH	*katG*	315	AGC → ACC	3,560
INH	*katG*	315	AGC → AAC	225
INH	*katG*	463	CGG → CTG	715
INH	*inhA*	−15	C → T	638
INH	*fabG1*	−15	C → T	128
INH	*ahpC*	11	CCG → CCG	85
RFP	*rpoB*	531	TCG → TTG	1883
RFP	*rpoB*	450	TCG → TTG	706
RFP	*rpoB*	526	CAC → GAC	238
EMB	*embB*	306	ATG → GTG	418
EMB	*embB*	306	ATG → ATA	165
QS	*gyrA*	94	GAC → GGC	894
QS	*gyrA*	94	GAC → GCC	202
QS	*gyrA*	90	GCG → GTG	560
QS	*gyrA*	95	AGC → ACC	244
SM	*rpsL*	43	AAG → AGG	1,163
SM	*rpsL*	88	AAG → AGG	212
SM	*rrs*	514	A → C	80
SM	*rrs*	1,401	A → G	76
SM	*rrs*	1,484	G → T	97
PZA	*pncA*	226	ACT→CCT	52
PZA	*pncA*	395	GGT → GCT	34

**Figure 5:**
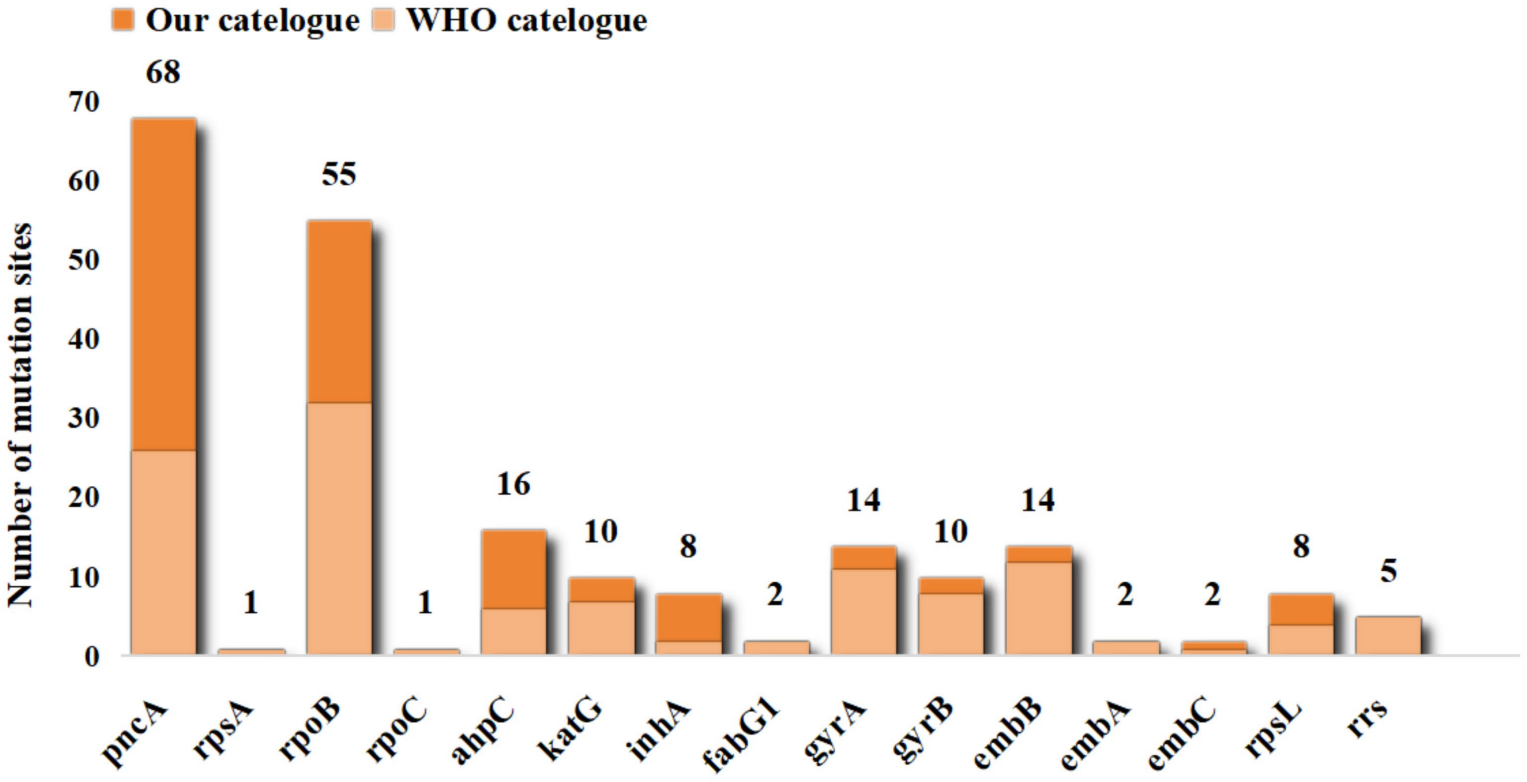
Number of candidate resistance genes associated with the WHO catalogue. *rpsA*, *rpoC*, *fabG1*, *embA*, and *rrs* all appear in the WHO catalogue, so there is only one dataset.

We further compared the mutation profile of drug resistance genes identified through our analysis with the catalogue published by the World Health Organization (hereinafter referred to as the WHO catalogue). Among the 215 candidate mutation sites, 121 were also documented in the WHO catalogue. Specifically, 32 mutation sites in our *rpoB* mutation profile were present in the WHO catalogue. For *pncA*, 26 mutation sites were recorded in the WHO catalogue. In contrast, only one synonymous mutation was documented in *rpsA.* Among the candidate genes associated with isoniazid resistance (INHR), *katG* and *ahpC* had 7 and 6 mutation sites listed in the WHO catalogue, respectively, whereas *kasA* was not included. The genes *rpsA*, *rpoC*, *fabG1*, *embA*, and *rrs* associated with drug resistance in our catalogue are all documented by the WHO (Figure 5). Given that the WHO catalogue is globally recognized as the authoritative reference for mutations related to tuberculosis drug resistance, the 96 mutation sites in our catalogue that the WHO did not document are considered novel mutations associated with drug resistance.

### 3.6 Combination of drug resistance gene mutations

**Figure 6:**
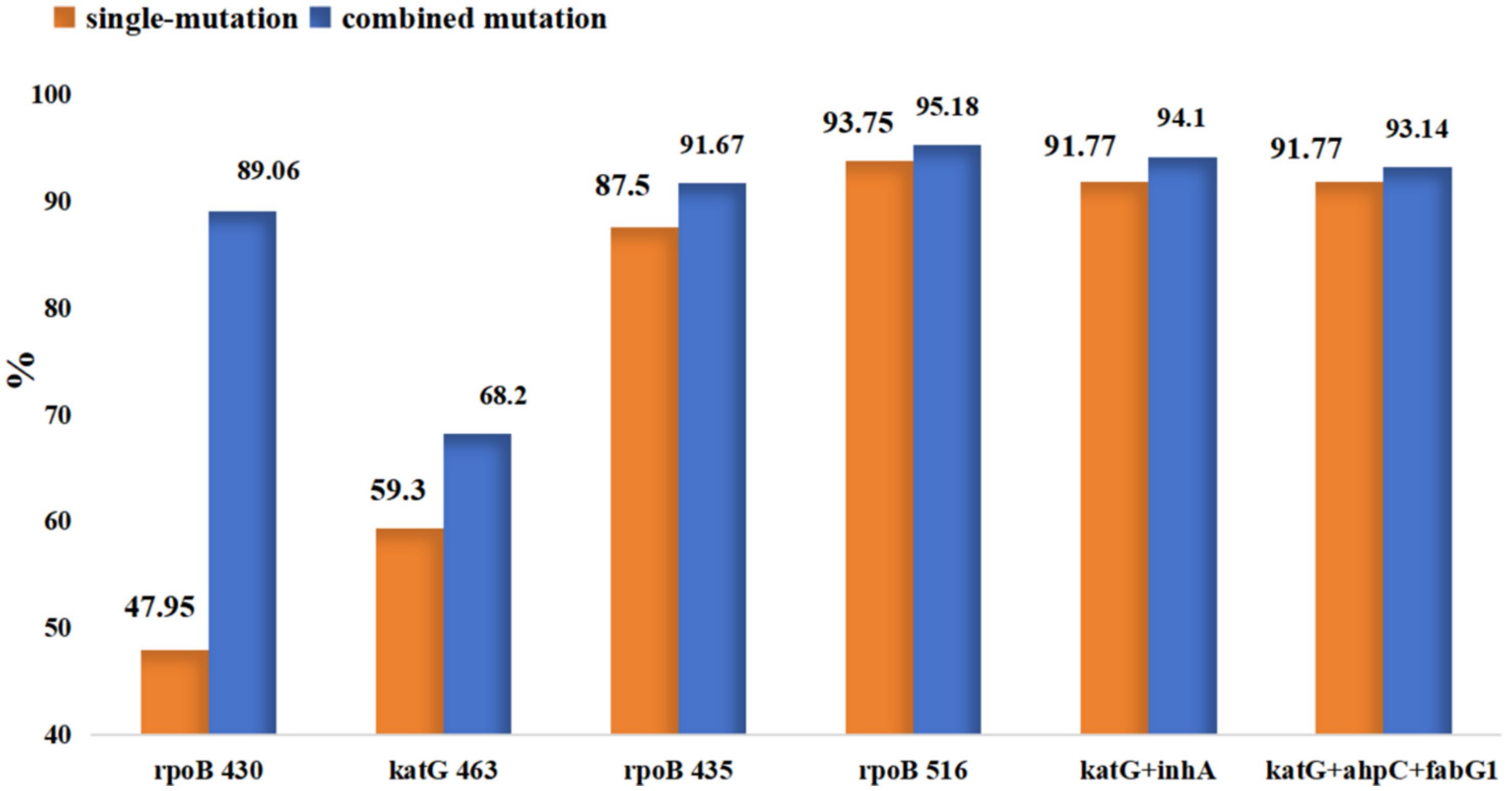
Positive predictive value of combined mutations in *rpoB* and *katG* for diagnosing RIF^R^ and INH^R^.

Current research findings indicate that drug resistance in many strains is not solely attributable to mutations in individual genes or loci but rather results from combined mutations involving multiple genes or sites. In this study, a total of 262 instances of combined mutations were identified, primarily involving mutations within different genes conferring resistance to the same drug or at different sites within the same gene (Supplementary Table 6). Among combined mutations associated with the same drug, those involving RIF and INH were the most frequent. For RIF, the predominant pattern consisted of *rpoB* + *rpoB* combined mutations. INH exhibited diverse mutational combinations, mainly comprising *katG* + *katG* and *katG* + inhA. Regarding mutations across different drugs, combined mutations were only observed between RIF and INH, primarily in the forms of *rpoB* + *katG* and *rpoB* + inhA. Although a substantial number of *pncA* mutations were identified in this study, only two combined mutations involving this gene were detected. Furthermore, combined mutations across three or four sites—such as *rpoB* + *katG* + inhA and *katG* + *inhA* + *ahpC* + *ndh*—were also identified in some resistant strains. The patterns of combined mutations among drug resistance genes are summarized in Table 3. Subsequently, we further analyzed several mutation sites in *rpoB* and *katG* and found that the PPV increased when combined mutations occurred compared to single-site mutations. The PPV for the *rpoB* 516 site increased from 93.75 to 95.18%, while that for the *rpoB* 430 site rose from 47.95 to 89.06%. For *katG* 463, the PPV increased from 59.30 to 68.20%. Additionally, combined mutations in different candidate genes associated with isoniazid resistance (INH^R^), such as *katG* + *inhA* and *katG* + *ahpC* + *fabG1*, increased the PPV from 92.83 to 94.84 and 93.41%, respectively (Figure 6).

**Table 3:** Patterns of combined mutations in candidate drug resistance genes.

Drug	Mutational combination	Total
RIF + RIF	*rpoB* + *rpoB*	81
	*rpoB* + *rpoC*	4
	*rpoB* + *rpoB* + *rpoB*	7
	*rpoB* + *rpoB* + *rpoB* + *rpoB*	1
RIF + INH	*rpoB* + *katG*	15
	*rpoB* + inhA	5
	*rpoB* + *rpoB* + *katG*	6
	*rpoB* + *katG* + inhA	1
INH + INH	*katG* + *katG*	20
	*katG* + inhA	16
	*katG* + *ahpC*	8
	*katG* + *fabG1*	8
	*katG* + *katG* + *katG*	4
	Others^*^	20
QS + QS	*gyrA* + *gyrA*	10
	*gyrA* + *gyrB*	23
	*gyrB* + *gyrB*	5
EMB + EMB	*embB* + *embB*	8
	*embB* + *embA*	7
	*embB* + *embC*	2
SM + SM	*rrs* + *rrs*	1
	*rrs* + *rpsL*	7
	*rpsL* + *rpsL* + *rrs*	1
PZA + PZA	*pncA* + *pncA*	2
Total	–	262

^*^Due to the complexity of the combined mutation patterns of resistance genes in INH, only the major binding patterns are listed.

## 4 Discussion

This study provides an updated overview of the current status of drug resistance in *Mycobacterium tuberculosis* in China. For the first time, a comprehensive analysis was conducted on drug resistance and genetic mutation profiles of 55,388 clinical MTB isolates collected from 27 regions across China, including 7,848 MDR-TB strains. Our study examined regional and temporal distribution patterns of these strains, and the results reveal a serious situation of drug-resistant tuberculosis in China, characterized by complex and diverse resistance profiles. Furthermore, we identified multiple novel mutation sites in candidate genes that are potentially associated with drug resistance but have not been previously documented by the WHO. Therefore, there is an urgent need in China to further investigate the mechanisms of drug resistance, rationally apply molecular detection technologies, and develop new anti-tuberculosis drugs that target novel mechanisms or unexplored pathways to address the increasingly complex landscape of drug resistance.

The 55,388 MTB strains included in the study, spanning a 20-year period, may reflect the characteristics of TB drug resistance development in China. The overall strain resistance rate was 27.22%, which was slightly higher than the resistance rate of retreatment patients reported in the 2012 national survey (25.6%), but still lower than the resistance rates in high-burden regions such as India (58.4%) and Russia (32.5%) (World Health Organization, 2023; Lohiya et al., 2020; Ismail et al., 2018). Among all DR-TB strains, INH (27.67%) and RIF (25.33%), the first-line anti-TB drugs, had the highest resistance rates, which increased compared with the 2007 Chinese National Drug-Resistant Tuberculosis Survey (Lohiya et al., 2020). Moreover, the resistance rates of EMB, SM, and PZA were 6.19, 11.55, and 8.63%, respectively. Notably, the resistance rate of PZA differed significantly from those of Thailand (49.0%) (Jonmalung et al., 2010) and Brazil (45.7%) (Bhuju et al., 2013). In addition, the resistance rate of second-line anti-tuberculosis drugs, especially QS, was higher than that of the first three and the results of the 2007 national survey (19.6%), but lower than the results of the Hameed et al. survey on resistance (73%) (Hameed et al., 2019). The observed differences in resistance rates indicate that the strategy of standardizing TB drug use has some positive effects. However, there are still problems, such as overuse of anti-tuberculosis drugs or unregulated treatment, as far as the current situation in China is concerned.

The MTB isolates in this study were obtained from multiple regions across China. Geographically, the highest number of isolates was collected from the eastern and western regions, which may be attributed to their relatively well-developed economic and healthcare infrastructure, facilitating more extensive strain surveillance and collection efforts. However, it is noteworthy that the drug resistance rates of MTB in these two regions were lower than those observed in the northeastern region. The drug resistance rate detected in the northeast exceeded both the nationally reported average and the levels documented in the survey by Wang et al. (2023). This phenomenon may be associated with historically irregular treatment practices and geographical differences in the prevalence of specific MTB genotypes, such as the Beijing genotype, which is potentially linked to higher drug resistance (Xu et al., 2017). The isolates included in this study cover a time span from 2002 to 2024. With the exception of INH^R^ strains, a distinct turning point in resistance rates was observed for all other types of DR-TB strains in 2019. For instance, limited human and material resources, coupled with the social stigma attached to tuberculosis patients due to coughing as a symptom, may lead individuals to conceal their condition from others and delay seeking medical care. This situation has been exacerbated by the COVID-19 pandemic, ultimately resulting in a reduction in the sources of bacterial strains (Bonadonna et al., 2017). According to data on drug-resistant strains acquired through the S-strategy, resistance rates to most anti-tuberculosis agents have shown an increasing trend in the past. Overall, however, the upward trajectory exhibited signs of moderation after the COVID-19 pandemic was brought under control, which aligns with global trends reported in the World Tuberculosis Report (World Health Organization, 2024). Considering both the geographical and temporal distribution characteristics of tuberculosis in China, we emphasize the need to further expand monitoring coverage, strengthen active screening, and accelerate the production and dissemination of national tuberculosis drug resistance surveillance reports. These steps are essential to providing timely and reliable data support for public health policy-making.

Compared to conventional DST, molecular detection methods based on pathogen nucleic acids, such as PCR amplification (Luo et al., 2019), whole-genome sequencing (WGS) (Wang et al., 2022), and line probe assays (Wang et al., 2021), offer improved prediction of drug resistance by leveraging limited known mutations and extensive catalogs of genetic variations (Wan et al., 2020). However, before investigating the association between genetic mutations and drug resistance, synonymous mutations and lineage-specific variations, such as *gyrA* Gly95Ala, which are recognized as unrelated to resistance, should be excluded (Siddiqi et al., 2002). In this study, we identified several synonymous mutations occurring exclusively in drug-resistant strains, including *inhA* 145 and *rpoB* 531, which we report here for the first time. Nevertheless, further strain identity information is required to determine whether these are linked to specific bacterial lineages. Among all synonymous mutations, the most frequently observed was *rpsA* 636 CGA → CGC, which is also documented in the WHO catalog. Notably, its PPV in our study (66.02%) was higher than that reported by the WHO (39.85%) (World Health Organization, 2023). Most of these strains were isolated from Henan Province, China, suggesting that the high frequency of this synonymous mutation in *rpsA* may be related to regional strain variations or other unknown factors. Additionally, the synonymous mutation *rpoB* 1,075 GCT → GCC was also frequently detected in DR-TB strains. Although lineage information for these strains was unavailable, our findings align with those reported by Wan et al. (2020) indicating that this mutation is closely associated with the Beijing genotype of MTB. The identification of synonymous mutations may contribute to a more accurate prediction of MTB drug resistance. Integrating such mutations with strain genotypic identity could provide further insights into the epidemiology of MTB.

Among the six anti-tuberculosis drugs in this study, INH and PZA are structurally simple. However, the resistance mechanisms are extremely complex, especially INH, which involves a wide variety of resistance genes with high mutation rates. Multiple previous studies have reported resistance genes associated with INH within biosynthetic networks and pathways (Unissa et al., 2016; Liu et al., 2019). *katG*, *ahpC*, inhA, and *fabG1* mutations predominated in this study, with an overall mutation rate of 32.91%. Among strains with *katG* mutations, 93.03% were drug-resistant, a finding consistent with the results reported by Isakova et al. (2018) (91.2%). The predominant mutations in *katG* were 315AGC → ACC and 463CGG → CTG. However, the latter mutation was frequently observed in both resistant and susceptible strains, and existing evidence suggests that this mutation is more closely associated with the genetic lineage of the strain (Arjomandzadegan et al., 2011). Furthermore, this study newly identified *ahpC* 11CCG → CCG as a major mutation occurring exclusively in DR-TB strains, which differs from the predominant mutation at position −52 reported by Flores-Treviño et al. (2015). Previous studies have indicated that the mutation rate and specific loci of *ahpC* exhibit regional variations, with differing mutation frequencies observed even for the same locus across different provinces in China (Jagielski et al., 2015). Therefore, this locus could be considered a potential new target for investigating its correlation with INH^R^. To supplement the findings of Pei et al. (2024), who did not include an analysis of PZA-related loci, this study adopted broader criteria to investigate the characteristics of PZA^R^ mutations in China. The results were consistent with previous reports (Tan et al., 2014), showing a high *pncA* mutation rate with widely dispersed mutation sites. In this study alone, 215 distinct mutation types were identified, primarily *pncA* 226ACT → CCT and 395GGT → GCT.

The chemical structures of RIF, EMB, SM, and QS are relatively complex. However, the candidate genes associated with drug resistance are predominantly concentrated in several hotspot loci where mutations frequently occur. The mutation rate for RIF was 28.98%, with mutations in DR-TB strains accounting for 97.03%—a figure higher than that reported by Yu et al. (2022) (90.00%). This discrepancy may be attributed to the fact that Zeng et al. (2021) utilized WGS on strains from a single region. In contrast, the present study integrated results from multiple molecular detection methods, ultimately identifying a larger number of strains, which may have led to an elevated mutation rate. Consistent with previous findings, the primary mutation sites in *rpoB* in this study were at positions 531 (38.16%) and 526 (15.21%). Additionally, a novel mutation at 531TCG → TGG was identified. EMB occurs mainly with the *embB*306 mutation, in which the amino acid Met → Ile change exists in three mutation patterns: ATG → ATT, ATG → ATC, and ATG → ATA. Furthermore, *embA* and *embC* also encode arabinosyltransferases, thus contributing to EMB^R^ (Khosravi et al., 2019). Examples include the *embC* 270ACC → ATC in conjunction with *embB* mutations, as well as *embA* C-12 T and C-16G occurring together with *embB* mutations. Among all mutated strains, the SM mutation rate was 10.07%, with DR-TB strains comprising 96.63% of these. The candidate genes for SM mutations were relatively stable and concentrated, primarily occurring in *rpsL* and *rrs*. Mutations in *rpsL* were mainly located at 43AAG → AGG and 88AAG → AGG, while those in *rrs* were concentrated at 514A → C and 1401A → G, consistent with both domestic and international studies (Chen et al., 2012; Smittipat et al., 2016). QS is a critical drug in the treatment of MDR-TB. In China, there is a high prevalence of pre-diagnostic exposure to QS among tuberculosis patients. In recent years, irregular usage and easy accessibility have contributed to a rising rate of QS resistance (Migliori et al., 2012). In this study, among strains with QS mutations, 98.34% were found to be drug-resistant. High-frequency mutations occurred mainly in *gyrA* and *gyrB*. Similar to the findings of Yin and Yu (2010), although most QS^R^ strains carried mutations at position 94 of *gyrA*, the mutation patterns were diverse. The primary mutation observed was 94GAC → GGC, along with six other mutation patterns in the 94 locus. By monitoring the emergence and prevalence trends of these mutations within populations, it is possible to tailor individualized drug dosages based on molecular diagnostic results.

MTB strains often employ multi-locus combined mutation strategies, in addition to accumulating single-gene mutations, to enhance drug resistance levels or compensate for fitness costs under antimicrobial pressure (Dookie et al., 2018). For instance, in RIFR, co-occurring mutations at codons 533, 531, and 516 of the rpoB gene, along with other sites, significantly increase the minimum inhibitory concentration (MIC) (Nusrath Unissa et al., 2016). Concurrent mutations in *rpoC* and *rpoB* often exert compensatory or modifying effects on *the phenotypes of rpoB mutations*, further refining the resistance profile (Farhat et al., 2013). In the context of INH^R^, combined mutations involving the *katG*, inhA, and *ahpC* genes frequently lead to higher-level resistance (Jagielski et al., 2014). Studies indicate that MTB often acquires *inhA* mutations first, followed by *katG* mutations, or establishes INH^R^ prior to developing RIF^R^, suggesting a close evolutionary linkage between these two resistance mechanisms. This pattern serves as an important molecular marker for diagnosing multidrug-resistant tuberculosis (MDR-TB) (Li et al., 2019). This study demonstrates that RIF and INH co-mutations are the most prevalent. RIF^R^ primarily involves multiple combined mutations within *rpoB* (e.g., 511 + 526, 516 + 533). Furthermore, all *ahpC* mutation sites identified in this study exhibited coupled mutations with *rpoB* at position 450. This phenomenon further corroborates the findings from the Russian population study, which showed that non-synonymous SNPs in *rpoC* are significantly more likely to occur in isolates harboring the *rpoB* mutation encoding p. Ser450Leu (Casali et al., 2014). INH^R^ exhibits more complex mutational patterns, including various combinations such as *katG* + inhA, *katG* + *ahpC*, and *ahpC* + inhA. Among these, the present study identified a coupled mutation within the *katG* gene (CAG295CAA + AGC315ACC). Whilst the mutation at position 315 (AGC315ACC, resulting in Ser315Thr) aligns with previously reported coupled mutations in India (Ramasubban et al., 2011), the mutation at position 295 in this study is synonymous (CAG295CAA, Gln295Gln), whereas the Indian study reported a missense mutation (CAG295CAC, Gln295His). This indicates that the two strains underwent distinct genetic events at position 295. Furthermore, several novel mutation patterns were identified in this study, including *pncA* + *pncA* and *rpsL* + *rpsL* + *rrs*. Whether these mutations significantly contribute to high-level resistance requires further validation with MIC data. In conclusion, the emergence of collaborative mutations suggests that MTB is undergoing active adaptive evolution, aimed at enhancing drug resistance and conferring a survival advantage through multiple genetic alterations. This evolutionary strategy not only improves the strain’s drug tolerance but also substantially complicates the clinical management and control of DR-TB.

Based on the information of MTB strains obtained from TB-related studies conducted in various regions, this study specifically analyzed the current status of the DR-TB epidemic in China and updated the catalogue of mutations associated with drug resistance. In addition to the major mutations already documented by the WHO, we identified several novel genetic loci that may be associated with drug resistance, such as *inhA*239, *ahpC*11, and *pncA*226, which also exhibited relatively high frequencies among resistant strains (*p* < 0.05). However, to determine the correlation between these loci and drug resistance, we need more information on the drug sensitivity results (e.g., MIC) of these strains as well as relevant validation experiments. Notably, although several high-frequency mutation sites listed in the WHO catalog were also present in our dataset, their prevalence profiles differed. For instance, the mutation rate at *ahpC*-52 was lower than that at positions −48 and 11. This discrepancy may reflect the WHO’s global data aggregation approach, suggesting that China may harbor a distinct mutation spectrum for DR-TB. Furthermore, among all mutations compiled in this study, the proportion of DR-TB strains is likely overestimated. This bias may arise from the fact that DR-TB isolates are more frequently reported and thus more likely to be included in such analyses. The purposeful launch of TB surveys in various regions has led to the proactive collection of strains that are already drug-resistant in their own right, as seen in studies by Wu et al. (2019) and Sun et al. (2022), among others. At the same time, not all candidate genes were analyzed in this study due to the difficulty of analysis, and most efflux pump genes and enzymes related to carbon source metabolism (e.g., Rv1258c and pckA) were not included in the candidate genes, which may have led to the prediction that some isolates with mutations were missed. Finally, because many of the original studies did not report strain typing information, and if this had been used as an inclusion criterion, it would have drastically reduced the amount of data available. The present study, after careful consideration, failed to systematically analyze the association between *Mycobacterium tuberculosis* strain profiles and specific drug resistance mutations. However, in future studies, we will endeavor to fill this knowledge gap in order to gain a deeper understanding of the strain context in which drug resistance arises.

Despite these limitations, in-depth analyses based on continuously updated MTB drug resistance surveillance data can still enhance our understanding of epidemiological trends and the development of DR-TB in China, making it possible to advance toward achieving 100% coverage of TB molecular diagnostic techniques by 2027. Our analysis of *Mycobacterium tuberculosis* strains suggests that China should adopt complementary diagnostic strategies tailored to local drug-resistance patterns and introduce faster, more sensitive molecular detection technologies. For instance, nanopore sequencing technology (Carandang et al., 2025) offers notable advantages in terms of rapid testing and portability. Such technologies should be effectively integrated with conventional drug susceptibility testing and validated in subsequent studies. As functional validation evidence similar to that generated in this study continues to accumulate, mutations supported by robust evidence should be incorporated into routine reporting protocols to inform individualized treatment planning directly. With ongoing technological advancement and increasing methodological maturity, these approaches will provide critical support for diagnosing and treating drug-resistant tuberculosis, thereby helping to alleviate the burden of drug-resistant tuberculosis in China.

## Data Availability

The original contributions presented in the study are included in the article/Supplementary material, further inquiries can be directed to the corresponding author.

## Supplementary material

Supplementary Table 1 Source of 55388 MTB strains included in this study.

Supplementary Table 2 Regional distribution of strains included in this study.

Supplementary Table 3 Temporal distribution of strains included in this study (which have been differentiated according to different detection strategies).

Supplementary Table 4 Synonymous mutations occurring in candidate genes and intervals in this study.

Supplementary Table 5 Catalogue of mutations in drug resistance genes of MTB strains in this study.

Supplementary Table 6 Combined mutation profiles of different drug-resistant genes and intervals.

Supplementary Table 7 The sixteen candidate genes and intergenic regions analyzed for association with resistance acquisition.

Supplementary Table 8 Distribution of 15078 resistant strains obtained by different testing strategies.
