# Supplementary material for: Drug resistance profile of Mycobacterium tuberculosis in China: update until 2024
Source: Front Microbiol. 2025 Dec 9;16:1697490. doi: 10.3389/fmicb.2025.1697490 (PMC12722995; doi:10.3389/fmicb.2025.1697490)
Supplement: Supplementary file 1 [file Data_Sheet_1.zip › Supplementary data sheet/Supplementary Table 7 .docx]

**Table 1 The sixteen candidate genes and intergenic regions analyzed for association with resistance acquisition.**

| ****Drug**** | ****Gene**** | ****Tier**** | ****Effect**** |
| --- | --- | --- | --- |
| INH | *katG* | 1 | **Encodes catalase-peroxidase.** Acts as the **activator enzyme for INH**, converting the inactive prodrug into its bactericidal form. |
| INH | *inhA*(upstream includes all of *fabG1*) | 1 | **Encodes enoyl-acyl carrier protein reductase.** **Mutations in the coding region** or **overexpression in the promoter region**, as well as mutations in the active site, reduce the affinity between *inhA* and activated INH. ***fabG1*** **encodes an acyl carrier protein reductase.** It is co-transcribed with the inhA operon. |
| INH | ***ahpC*** | 1 | **Encodes alkyl hydroperoxide reductase.** Compensates for *katG* mutations, aiding the bacteria in resisting oxidative stress. |
| INH | *kasA* | NA | Encodes β-ketoacyl-ACP synthase, involved in mycolic acid biosynthesis. |
| RIF | *rpoB* | 1 | **Encodes the β-subunit of RNA polymerase.** **Mutations** alter the structure of the drug-binding target. |
| RIF | *rpoC* | 2 | **Encodes the β'-subunit of RNA polymerase.** Acts as a **compensatory mutation**, helping restore bacterial fitness and consolidating drug resistance. |
| EMB | *embB* | 1 | **Encodes an arabinosyltransferase.** Alters the biosynthesis of arabinan in the cell wall. |
| EMB | *embC* | 1 | **Encodes an arabinosyltransferase.** Homologous to *embB* and *embA*, collectively involved in cell wall synthesis. |
| EMB | *embA* | 1 | **Encodes an arabinosyltransferase.** Homologous to *embB* and *embC*; its mutations, particularly in the promoter region, are associated with EMB resistance. |
| SM | *rpsL* | 1 | **Encodes ribosomal protein S12.** Mutations alter the ribosome structure, preventing streptomycin binding and leading to **high-level resistance**. |
| SM | *rrs* | 1 | **Encodes 16S rRNA.** Mutations alter the streptomycin-binding site on the ribosome, resulting in resistance. |
| QS | *gyrA* | 1 | **Encodes the A subunit of DNA gyrase.** **Mutations** significantly reduce drug-enzyme binding affinity, leading to high-level resistance. |
| QS | *gyrB* | 1 | **Encodes the B subunit of DNA gyrase.** Mutations reduce drug-target affinity, resulting in resistance. |
| PZA | *pncA* | 1 | **Encodes pyrazinamidase.** Activates the prodrug PZA into the active form, pyrazinoic acid. Mutations cause enzyme inactivation, preventing drug activation. |
| PZA | *panD* | 1 | **Encodes aspartate decarboxylase—a** key enzyme in coenzyme A biosynthesis. Mutations affect the metabolic state of the bacterium, leading to PZA resistance. |
| PZA | *rpsA* | 2 | **Encodes ribosomal protein S1.** The active drug pyrazinoic acid binds to *rpsA* and inhibits the trans-translation process. |

NA=not available.
