# Supplementary material for: Drug resistance profile of Mycobacterium tuberculosis in China: update until 2024
Source: Front Microbiol. 2025 Dec 9;16:1697490. doi: 10.3389/fmicb.2025.1697490 (PMC12722995; doi:10.3389/fmicb.2025.1697490)
Supplement: Supplementary file 1 [file Data_Sheet_1.zip › Supplementary data sheet/Supplementary Table 8.docx]

**Table 1** Distribution of 15078 resistant strains obtained by different testing strategies.

| **Drug** | **Testing Strategy** | **Frequency** | **Total Frequency** |
| --- | --- | --- | --- |
| INHR | Single-Drug Testing | 1208 | 5721 |
|  | Multi-Drug Testing | 4513 |  |
| RFPR | Single-Drug Testing | 1057 | 5238 |
|  | Multi-Drug Testing | 4181 |  |
| QSR | Single-Drug Testing | 2486 | 4265 |
|  | Multi-Drug Testing | 1779 |  |
| SMR | Single-Drug Testing | 351 | 2387 |
|  | Multi-Drug Testing | 2036 |  |
| PZAR | Single-Drug Testing | 1624 | 1784 |
|  | Multi-Drug Testing | 160 |  |
| EMBR | Single-Drug Testing | 52 | 1280 |
|  | Multi-Drug Testing | 1228 |  |
| Total Frequency | — | 20675 | 20675 |
